# From Fresh to Aged: Dynamics of Lipid Stability, Fatty Acid Profile, and Meat Quality of Dry‐Aged Dorsal Epaxial Muscle of Mahi‐Mahi (*Coryphaena hippurus*)

**DOI:** 10.1111/1750-3841.71074

**Published:** 2026-05-06

**Authors:** Rômulo D. Lopes, Sérgio B. Mano, André L. M. Souza, Jane S. M. Castro, Lévison C. Cipriano, João P. A. D. Ultra, Adriano G. Cruz, Renata S. L. R., Celso F. Balthazar, Eliane T. Mársico

**Affiliations:** ^1^ Veterinary Medicine Faculty Fluminense Federal University Niterói, State of Rio de Janeiro Brazil; ^2^ State Department of Agriculture, Livestock and Supply SEAPA Rio de Janeiro, State of Rio de Janeiro Brazil; ^3^ Portal da Saude do RJ Rio de Janeiro, State of Rio de Janeiro Brazil; ^4^ Food Department of the Federal Institute of Education Science and Technology of Rio de Janeiro State of Rio de Janeiro Brazil

**Keywords:** dolphinfish, dry‐aging, fatty acid profile, lipid oxidation, meat quality, texture

## Abstract

This study investigated the physicochemical and nutritional changes in mahi‐mahi (*Coryphaena hippurus*) dorsal epaxial muscle during a 42‐day dry‐aging period (3°C, 83% RH, 0.8 m/s laminar airflow). While dry‐aging is well‐established to enhance tenderness in slaughter animals’ meat, its application to seafood, particularly species with high polyunsaturated fatty acid (PUFA) content, remains scientifically underexplored due to risks of oxidative degradation. Fresh specimens were processed within 2 hours post‐capture and analyzed at 0, 15, 27, and 42 days. Moisture decreased from 76.9% to 61.5%, while protein content increased from 20.9% to 35.4% due to concentration and proteolysis. Lipid oxidation of thiobarbituric acid reactive substances increased at 0.56 mg MDA/kg by day 27, indicating substantial degradation of PUFAs. Hardness initially decreased by 76% at day 15, and then partially recovered due to protein aggregation and moisture loss. Color parameters shifted toward darker (*L* decrease) and greenish (*a* decrease) tones, reflecting myoglobin oxidation. The fatty acid exhibited early lipolysis (increased oleic acid C18:1 *ω*‐9) followed by oxidative degradation. Nutritional indices (atherogenic index, thrombogenic index, hypocholesterolemic/hypercholesterolemic fatty acid ratio) improved up to day 27, decreasing thereafter. These findings suggested that dry‐aging mahi‐mahi for 27 days optimized quality by balancing texture development with acceptable oxidative stability, providing a scientific basis for premium dry‐aged seafood products.

**Practical Applications:**

The study demonstrated that mahi‐mahi can be commercially dry‐aged for 5–27 days (3°C and 83% RH) to enhance texture. This process concentrated proteins and developed desirable sensory attributes through controlled enzymatic activity and moisture loss while maintaining nutritional quality before oxidative rancidity increased. By adopting this method, seafood producers could offer a premium, value‐added product aligned with culinary additive‐free trends, such as novel foods. Routine monitoring of lipid oxidation and moisture loss was recommended to ensure consistent quality. This research provided a scientifically validated approach for product diversification and market differentiation within the seafood industry.

## 1 Introduction

Fish is a nutritionally valuable food, rich in polyunsaturated fatty acids (PUFAs), high‐quality proteins, and essential minerals (Singh et al. 2025). However, its high moisture content and labile lipids render it highly susceptible to spoilage via microbial action, enzymatic degradation, and oxidative rancidity (Zhuang et al. 2021; Cheng et al. 2024). Growing consumer demand for natural, additive‐free preservation and novel culinary experiences has recently renewed interest in traditional techniques like dry‐aging for seafood (Panebianco et al. 2024; Pushparaj et al. 2025). While extensively studied in terrestrial meats such as beef for enhancing tenderness (Savas et al. 2024), the application of dry‐aging to fish remains scientifically underexplored.

Dry‐aging involves the controlled storage of unpackaged meat under regulated temperature, humidity, and airflow (Pushparaj et al. 2025). This process induces complex biochemical transformations, including proteolysis, moisture loss, and oxidative reactions affecting color and aroma (Álvarez et al. 2021; Chmiel 2024). In fish, these oxidative processes can accelerate PUFA degradation, thereby diminishing nutritional value (Shahidi and Hossain 2022; Shaviklo 2023). Thus, controlling lipid oxidation is essential for developing high‐quality dry‐aged fish products.

Mahi‐mahi (*Coryphaena hippurus*), or dolphinfish, is a globally important commercial and recreational species prized for its firm texture (Moltó et al. 2020; Costa et al. 2021). Its lean‐to‐moderate fat content and robust muscle structure suggest suitability for extended aging without structural collapse (Moltó et al. 2020). Despite this potential, no comprehensive study has systematically tracked the physicochemical and oxidative changes in mahi‐mahi throughout a prolonged dry‐aging process. In particular, data on the evolution of its fatty acid profile conditions are lacking as a key determinant of nutritional quality and stability.

The dry‐aging parameters selected for this study, temperature maintained at 3°C (±0.5°C), relative humidity at 83% (±2%), and laminar airflow at 0.8 m/s (±0.2 m/s), were strategically chosen to balance enzymatic aging processes with microbial control over an extended 42‐day period. These conditions align with established recommendations for beef dry‐aging (0–4°C, 75%–80% RH, 0.5–2 m/s airflow), which successfully support aging for 28–55 days while developing desirable flavor and texture (Ribeiro et al. 2024; Kang et al. 2022). Previous work on rainbow trout dry‐aged under similar conditions (∼3°C–3.6°C, ∼78% RH) demonstrated acceptable microbiological quality up to 10 days, with spoilage indicators emerging by day 14 (Panebianco et al. 2024). However, no studies have characterized fish dry‐aged beyond 14 days, leaving a significant knowledge gap regarding extended aging.

Therefore, this study addressed this gap by investigating physicochemical changes in mahi‐mahi over 42 days of dry‐aging. The objectives were to (1) characterize the progression of lipid oxidation and its impact on fatty acid profile and nutritional indices; (2) correlate these changes with modifications in texture and color; and (3) identify an optimal aging duration that maximizes sensory improvements while minimizing quality degradation. The findings aimed to provide a scientific basis for standardizing dry‐aging protocols for premium seafood, offering the industry a method to add value and meet evolving consumer trends.

## 2 Materials and Methods

### 2.1 Sample Collection and Preparation

Six specimens of mahi‐mahi (*C. hippurus*), between 8 and 10 kg each, were procured from artisanal fisheries in Rio de Janeiro, Brazil. Immediately post‐harvest, the fish were eviscerated in aseptic conditions and transported to the laboratory in a clean cooler containing filtered ice under chilled conditions (−1°C/+1°C) to minimize metabolic degradation. In the lab, fish specimens were aseptically clean, and dorsal epaxial muscle was prepared for an aging process, which was completed within 2 hours of capture, after which the specimens were transferred to a benchtop climate‐controlled dry‐aging chamber of 50 L internal volume, dimensions 400 × 400 × 400 mm (WEW‐64L‐TA; Wewon Environmental Chambers Co., Ltd., China). One specimen was reserved as a fresh control sample (Day 0) and analyzed without aging. The remaining five specimens underwent controlled dry‐aging, suspended individually within the chamber to optimize air circulation and ensure consistent exposure to environmental parameters.

### 2.2 Dry‐Aging Process

The fish specimens were dry‐aged for 42 days in a commercial refrigerated chamber under precisely controlled conditions: temperature maintained at 3°C (±0.5°C), relative humidity at 83% (±2%), and a laminar airflow of 0.8 m/s (±0.2 m/s). Environmental parameters were continuously monitored using digital data loggers and verified twice a day (at the beginning and end of the working day) to ensure process uniformity. Also, samples were collected at fixed, predetermined time intervals performed at 0‐, 15‐, 27‐, and 42‐day intervals during the experimental period. At each sampling point, a representative amount of meat (approximately 100 g each) was aseptically excised from the dorsal epaxial muscle of each fish. Samples were immediately vacuum‐packaged using a commercial double‐chamber vacuum sealer (Modulare CAV 40; Conceito Vácuo, São Paulo) and flash‐frozen at −20°C in a blast freezer for long‐term preservation prior to subsequent physicochemical analyses.

### 2.3 Proximate Composition

The proximate composition analysis was conducted following standard protocols established by the Association of Official Analytical Chemists (AOAC 2005). Moisture content (AOAC 930.15) was quantified gravimetrically by oven‐drying samples for 3 hours at 105°C (±1°C) until constant mass was achieved, with measurements recorded to 0.1 mg precision. The moisture content was calculated using the formula:

(1) $$\mathrm{Moisture}\left(\%\right)=\left[\left(W_{1}-W_{2}\right)/\left(W_{1}-W_{0}\right)\right]\times 100$$

where *W*_0_ means the weight of the empty dish, *W*_1_ is the weight of the dish plus sample before drying, and *W*_2_ is the weight of the dish plus sample after drying.

Total protein content was determined using the micro‐Kjeldahl method (AOAC 984.13), where organic nitrogen was converted to ammonium sulfate via acid digestion, distilled, and titrated. The nitrogen content was subsequently converted to crude protein using the standard conversion factor of 6.25 for marine proteins. The crude protein content was calculated as follows:

(2) $$\mathrm{Crude}\mathrm{protein}\left(\%\right)=\left[\left(V-V_{0}\right)\times N\times 14.007\times CF/m\right]\times 100$$

where *V* means the volume of acid (HCl/H_2_SO_4_) used to titrate the sample (mL), *V*_0_ the volume for the blank (mL), *N* the normality of the acid, 14.007 the atomic weight of nitrogen, CF the conversion factor (6.25), and m the mass of the sample (g).

Lipid extraction was performed using a Soxhlet apparatus (AOAC 920.39) with petroleum ether (40°C–60°C boiling range) as the organic solvent, following complete drying of samples to prevent hydrolysis interference. Total lipid content was calculated from the weight difference between pre‐ and post‐extraction. The lipid content was determined as follows:

(3) $$\mathrm{Lipid}\left(\%\right)=\left[\left(W_{2}-W_{0}\right)/\left(W_{1}-W_{0}\right)\right]\times 100$$

where *W*_0_ means the weight of the dry extraction cup, *W*_1_ the weight of the cup plus the dry sample before extraction, and *W*_2_ the weight of the cup plus the extracted fat after solvent evaporation.

Ash content analysis (AOAC 942.05) involved combustion of organic matter in a muffle furnace at 550°C (±5°C) for 6 h, with crucibles cooled in a desiccator prior to gravimetric measurement. The ash content was calculated using the formula:

(4) $$\mathrm{Ash}\left(\%\right)=\left[\left(W_{2}-W_{0}\right)/\left(W_{1}-W_{0}\right)\right]\times 100$$

where *W*_0_ means the weight of the empty crucible, *W*_1_ the weight of the crucible plus sample before asking, and *W*_2_ the weight of the crucible plus ash after incineration.

All analyses were performed in triplicate to ensure methodological reliability.

### 2.4 Lipid Oxidation

Lipid oxidation was assessed by quantifying thiobarbituric acid reactive substances (TBARS), a renowned method from Sinnhuber and Yu (1958) and Buege and Aust (1978). This spectrophotometric assay measures malondialdehyde (MDA), a secondary lipid peroxidation product, formed during oxidative degradation of PUFAs. Briefly, 5.0 (±0.1 g) of homogenized fish tissue was mixed with 10 mL of 7.5% (w/v) trichloroacetic acid (TCA) containing 0.1% (w/v) ethylenediaminetetraacetic acid as a chelating agent. The mixture was centrifuged at 3000 × *g* for 10 min at 4°C. An aliquot (2 mL) of the supernatant was reacted with 2 mL of 0.02 M thiobarbituric acid (TBA) in a screw‐capped test tube. The reaction mixture was heated at 95 ± 1°C for 30 min in a water bath, then cooled in ice for 10 min to stop the reaction. The absorbance was measured at 532 nm against a blank (TCA‐TBA mixture) using a UV–Vis spectrophotometer (Model UV‐1800, Shimadzu, Japan). TBARS values were calculated from a standard curve of 1,1,3,3‐tetraethoxypropane (0–10 µM MDA equivalents) and expressed as mg MDA per kg of wet tissue weight.

### 2.5 Color Measurement

Color measurements were performed using a calibrated CM‐600D portable spectrophotometer (Konica Minolta Sensing, Inc., Tokyo, Japan) configured with d/8° measurement geometry, 10° observer angle, and D65 illuminant. Prior to each measurement session, the instrument was calibrated using the manufacturer's certified white calibration tile (*Y* = 94.1, *x* = 0.3158, *y* = 0.3323). Five random measurements were taken from the dorsal epaxial muscle region of each sample, maintaining the probe perpendicular to the muscle fiber orientation to ensure measurement consistency. Ambient light was controlled to 500 LUX, and sample surface temperature was maintained at room temperature (22±1°C) during measurements.

Color parameters were recorded in CIELAB color space, including L* (lightness), a* (red‐green chromaticity), and b* (yellow‐blue chromaticity). From these primary values, two derived color metrics (hue angle—h° and chroma—C*) were calculated according to Kuehni (1976)

(5) $$C^{*}=\sqrt{\left(a^{*2}+b^{*2}\right)}$$

(6) $$h^{\circ }=arctan\left(b*/a*\right)$$

### 2.6 Texture Profile Analysis

Texture profile analysis (TPA) was performed using a TA.XTplus texture analyzer (Stable Micro Systems Ltd., Godalming, UK) equipped with a 50 kg load cell and a 36 mm diameter cylindrical aluminum probe (P/36) (Liang et al. 2020). Briefly, uniform cubes measuring 2.0 × 2.0 × 2.0 cm (±0.1 cm) were excised from the dorsal epaxial muscle, with careful attention to maintaining parallel surfaces and consistent muscle fiber orientation relative to the compression axis. The analyzer was programmed to conduct a two‐cycle compression test with the following parameters: pre‐test speed of 1.0 mm/s, test speed of 1.0 mm/s, post‐test speed of 1.0 mm/s, target deformation of 40% original height, trigger force of 5 g, and a 5‐second pause between cycles. In addition, data acquisition was performed using Texture Exponent 32 software (v6.1.16.0), and all tests were conducted at a controlled temperature of 4°C (±1°C) to preserve sample integrity.

From the resulting force–time curves, seven fundamental texture parameters were quantified according to standard TPA methodology. Hardness was determined as the maximum peak force during the first compression cycle, expressed in Newton (N). Adhesiveness, measured in newton‐seconds (N·s), represented the negative force area corresponding to the work required to overcome sample‐surface stickiness. Springiness was calculated as the dimensionless ratio of sample recovery time between compressions. Cohesiveness was derived as the dimensionless ratio of work during the second compression to that of the first compression. Gumminess was computed as the product of hardness and cohesiveness (N), while chewiness was determined as the product of gumminess and springiness (N·mm). Resilience was quantified as the dimensionless ratio of upstroke to downstroke energy during the first compression cycle.

### 2.7 Fatty Acid Profile

Lipids were extracted from fish specimens using a cold solvent system consisting of methanol:chloroform:water (2:1:0.8, v/v/v). The extracted lipids were subsequently methylated under acidic conditions using a 10% methanolic–HCl solution according to Moura et al. (2024). For gas chromatographic analysis, 1 µL of the derivatized sample was injected into a gas chromatograph (Perkin Elmer, Waltham, MA, USA) equipped with a flame ionization detector (FID).

Separation of fatty acid methyl esters (FAMEs) was achieved using an Omegawax‐320 capillary column (30 m × 0.32 mm i.d., 0.25 µm film thickness; Supelco, Bellefonte, PA, USA) with ultra‐high‐purity helium as the carrier gas at a constant flow rate of 1.8 mL/min. The GC system operated with the injector port maintained at 260°C in split mode (1:20 split ratio, 10 psi), while the FID detector temperature was set at 280°C. The oven temperature program initiated at 110°C, followed by a rapid increase at 40°C/min to 233°C (2 min hold), then a gradual ramp of 1°C/min to 240°C, with a final isothermal period of 21 min.

Identification of individual FAMEs was accomplished by comparing retention times with those of a 37‐component FAME reference standard (Supelco 18919‐1AMP; Sigma‐Aldrich, St. Louis, MO, USA). Samples relative retention times and peak areas were calculated using the chromatography data system software. Quantification was performed by normalizing peak areas relative to the internal standard and applying response factors determined from the standard mixture calibration curves.

Lipid indices were calculated to evaluate the health‐related quality of the fatty acid profile: atherogenic index (AI), thrombogenic index (TI), and the hypocholesterolemic/hypercholesterolemic fatty acid ratio (H/H). These indices were determined using the following established equations (Santos‐Silva et al. 2002; Ulbricht and Southgate 1991):

(7) $$\mathrm{AI}=\left[C12:0+\left(4\times C14:0\right)+C16:0\right]/\left(\Sigma \mathrm{MUFA}+\Sigma \mathrm{PUFA}\right)$$

(8) $$\mathrm{TI}=\frac{\left[C14:0+C16:0+C18:0\right]}{[(0.5\times \Sigma \mathrm{MUFA})+(0.5\times \Sigma n-6\mathrm{PUFA})+(3\times \Sigma n-3\mathrm{PUFA})+\left(n-3/n-6\right)]}\;$$

(9) $$H/H=\left(C18:1n-9+\Sigma PUFA\right)/\left(C12:0+C14:0+C16:0\right)$$

### 2.8 Statistical Analysis

A total of six independent experiments were conducted, each using one fish specimen (*n* = 6). Individual specimens were considered as experimental units to account for biological variability and avoid results being biased by a single outlier. All analyses were performed in triplicate for each specimen to ensure methodological reliability, with results reported as mean values ± standard deviation. Data (proximate composition, lipid oxidation, color parameters, texture profile, fatty acid profile, and nutritional indices across the aging days: 0, 15, 27, and 42) were subjected to statistical analysis using one‐way analysis of variance (ANOVA) to evaluate significant differences across maturation time points. When ANOVA indicated significant effects (*p* < 0.05), Tukey's honestly significant difference (HSD) post‐hoc test was applied for pairwise comparisons between specific aging periods. The chosen significance threshold (*α* = 0.05) provided a 95% confidence level for all statistical inferences. Pearson correlation coefficients were calculated to evaluate linear relationships between lipid content and lipid oxidation (TBARS) across aging time points. Correlation strength was interpreted as weak (|*r*| < 0.3), moderate (0.3 ≤ |*r*| < 0.7), or strong (|*r*| ≥ 0.7), with significance set at *p* < 0.05. Statistical computations were performed using Statgraphics Centurion (version 19.4.02; Statgraphics Technologies, Inc., The Plains, VA, USA) with verification of assumptions including homogeneity of variance (Levene's test) and normality of residuals (Shapiro–Wilk test). Complementary data organization and preliminary analyses were conducted in Microsoft Excel (Microsoft 365 version 2308; Microsoft Corporation, Redmond, WA, USA). Effect sizes were calculated for significant findings to assess the practical importance of observed differences.

## 3 Results and Discussion

### 3.1 Proximate Composition Evaluation

Dry‐aging significantly altered the proximate composition of mahi‐mahi dorsal epaxial muscle over 42 days (*p* < 0.05, Table 1). Moisture content progressively decreased (*p* < 0.05) from 76.9% (Day 0) to 61.5% (Day 42), with the most pronounced loss (*p* < 0.05) occurring between Days 27 and 42. This dehydration trend aligned with dry‐aging studies on dry‐cured fish (Hu et al. 2025; Wang et al. 2024), where controlled evaporation drove moisture loss. The accelerated dehydration in later stages suggested prolonged aging exacerbated moisture migration, which is a critical factor in lean fish to prevent excessive yield loss and textural hardening (Oyinloye and Yoon 2025).

**TABLE 1:** Composition values of dry‐aged dorsal epaxial muscle of mahi‐mahi (*Coryphaena hippurus*) (*n* = 6).

Aging day	Moisture (%)	Protein (%)	Lipids (%)	Ashes (%)
D0	76.9^a^ ± 0.18	20.9^d^ ± 1.28	1.3^c^ ± 0.63	1.4^b^ ± 0.61
D15	73.0^b^ ± 1.9	24.4^d^ ± 1.78	2.1^a^ ± 0.08	1.5^b^ ± 0.06
D27	68.2^c^ ± 1.1	29.2^b^ ± 2.67	1.3^c^ ± 0.04	2.0^a^ ± 0.27
D42	61.5^d^ ± 1.9	35.4^a^ ± 1.45	1.6^b^ ± 0.71	2.2^a^ ± 0.24

*Note*: Different letters in the same column means significant differences between aging days by Tukey's honestly significant difference (HSD) post‐hoc test (*p* < 0.05) followed by ±standard deviation (SD).

Conversely, protein content increased markedly (*p* < 0.05) from 20.9% (Day 0) to 35.4% (Day 42), primarily due to moisture concentration. The sharpest increase (*p* < 0.05) occurred between Days 15 (24.4%) and 27 (29.2%), coinciding with mid‐stage proteolytic activity. Endogenous enzymes such as calpains and cathepsins likely contributed to protein breakdown, releasing soluble peptides and free amino acids that enhanced nutritional and functional development (Zhong et al. 2025). A similar protein concentration trend was observed in dry‐cured fish (Hu et al. 2025; Wang et al. 2024).

Lipid content fluctuated throughout aging, increasing initially from 1.3% (Day 0) to 2.1% (Day 15, *p* < 0.05), then declining to 1.3% (Day 27, *p* < 0.05), with a slight rise to 1.6% (Day 42, *p* < 0.05). This nonlinear pattern revealed a moderate negative correlation (*r* = −0.545) between lipid content and TBARS values (Section 3.2), establishing a mechanistic link between lipid degradation and oxidative processes. The inverse relationship, where lipid content declined precisely when oxidation products peaked, confirmed that oxidative degradation, rather than simple mobilization, drove lipid loss during mid‐aging (Tatiyaborworntham et al. 2022).

The initial increase at Day 15 (from 1.3% to 2.1%) occurred while TBARS remained low (0.21 mg MDA/kg), suggesting this phase was dominated by early lipid modifications or concentration effects rather than oxidative consumption. However, by Day 27, the sharp TBARS peak (0.56 mg MDA/kg) coincided with lipid content returning to baseline (1.3%), indicating active oxidative depletion of PUFAs (Section 3.5) (Zhou et al. 2022). The slight lipid rise at Day 42 (1.6%, *p* < 0.05), despite sustained high TBARS (0.53 mg MDA/kg), reflected the amplifying effects of cumulative moisture loss (from 76.9% to 61.5%), concentrating remaining lipids, which outweighed further oxidative depletion of oxidizable substrates (Fitri et al. 2022). This concentration‐amplification mechanism, also observed in dry‐cured Wuchang fish (Hu et al. 2025) and dry‐cured Spanish mackerel (Wang et al. 2024), explains how moisture dynamics interact with oxidative processes to shape final lipid profiles.

Ash content remained stable (*p* > 0.05) between Days 0 (1.4%) and 15 (1.5%) but increased significantly (*p* < 0.05) by Day 27 (2.0%) and Day 42 (2.2%), likely due to mineral concentration from moisture loss.

Beyond autolytic enzymes, surface microbial communities (e.g., *Pseudomonas* spp. and yeasts) may contribute to compositional changes by secreting exogenous lipolytic and proteolytic enzymes, potentially amplifying hydrolysis of triglycerides and myofibrillar proteins (Aqel et al. 2023; Basso et al. 2017). This microbial activity can influence the release of free fatty acids and peptides, contributing to characteristic dry‐aged products (Liu et al. 2025). The suitability of lean fish for such processes is increasingly recognized, with recent work on monkfish (*Lophius piscatorius*) confirming its potential for dry‐aging precisely due to its compositional profile (Thamsborg et al. 2025).

### 3.2 Lipid Oxidation Evaluation

**FIGURE 1:**
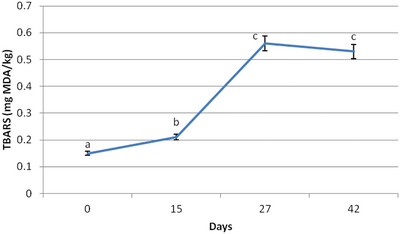
Lipids oxidation evaluation during 42 dry aging days of dorsal epaxial muscle of mahi‐mahi (*Coryphaena hippurus*) (*n* = 6). Error bars represent the standard deviation of the mean (±0.01). Different letters means significant differences between aging days by Tukey's honestly significant difference (HSD) post‐hoc test (*p* < 0.05).

Lipid oxidation in mahi‐mahi dorsal epaxial muscle during dry‐aging was assessed via TBARS (Figure 1). Fresh muscle (Day 0) exhibited low baseline oxidation (0.15 mg MDA/kg), confirming initial freshness. By Day 15, TBARS increased to 0.21 mg MDA/kg (*p* < 0.05), indicating early oxidative changes within acceptable quality limits (Mittakos et al. 2025). A sharp rise occurred by Day 27 (0.56 mg MDA/kg; *p* < 0.05), representing a critical transition point where cumulative oxidative stress overwhelmed endogenous antioxidant defenses due to depletion of fat‐soluble antioxidants, moisture loss concentrating pro‐oxidant factors, and structural membrane breakdown exposing oxidation‐prone phospholipids (Cheng et al. 2024).

The timing of this oxidation peak directly explains the concurrent decline in total lipid content (1.3% at Day 27) and the selective depletion of unsaturated fatty acids (Section 3.5). This cross‐validation across multiple analytical endpoints strengthens the mechanistic interpretation that oxidative processes, not merely lipolytic release, governed fatty acid dynamics (Zhou et al. 2022). Thereafter, TBARS values stabilized (0.53 mg MDA/kg at Day 42; *p* > 0.05 vs. Day 27), indicating either depletion of oxidizable PUFA substrates or formation of secondary oxidation products less reactive in the TBARS assay. The stabilization phase, despite continued moisture loss, suggests that oxidative kinetics were substrate‐limited rather than environmentally limited, a distinction important for optimizing aging duration (Pretto et al. 2024; Thamsborg et al. 2025).

The moderate increase by Day 15 reflected initial autoxidation of unsaturated fatty acids, particularly eicosapentaenoic acid and docosahexaenoic acid, which are abundant in marine species and highly susceptible to radical‐mediated oxidation (Cheng et al. 2024; Gonçalves et al. 2021). Pro‐oxidant factors such as lipoxygenase activity or heme iron released from myoglobin might also contribute to lipid oxidation (Ribeiro et al. 2021). Compared to beef, which exceeded 0.5 mg MDA/kg by Day 14 (Buege and Aust 1978), *C. hippurus* maintained lower TBARS at this stage, possibly due to lower initial fat content or endogenous antioxidants (e.g., tocopherols, astaxanthin) delaying oxidation (Cheng et al. 2024; Van Nieuwerburgh et al. 2005).

The delayed onset of oxidation beyond Day 15 could be attributed to the initial protective effect of the fish's endogenous antioxidant system. Pelagic species like mahi‐mahi accumulate fat‐soluble antioxidants (*α*‐tocopherol and astaxanthin) through their diet, which act as free radical scavengers (Bell et al. 2000). Enzymatic antioxidants such as glutathione peroxidase and superoxide dismutase might also retain residual post‐mortem activity, mitigating oxidative stress (Jomova et al. 2023). The sharp TBARS increase after Day 15 likely corresponded to depletion of these defenses, allowing pro‐oxidants to dominate and promote uncontrolled oxidation of highly unsaturated membrane phospholipids (Aubourg 2023; Korczowska‐Łącka et al. 2023).

The peak at Day 27 marked a transition point, probably driven by cumulative oxidative stress from prolonged oxygen exposure, moisture loss concentrating pro‐oxidants, and antioxidant depletion (Ribeiro et al. 2021). Structural breakdown of muscle membranes might also release oxidation‐prone phospholipids (Cheng et al. 2024). The subsequent decline in lipid content after Day 15 (Table 1) supported oxidative degradation as a primary driver of lipid loss, consistent with TBARS correlations in lamb (Gürbüz et al. 2022).

Oxidation kinetics were influenced by dry‐aging parameters (83% RH, 0.8 m/s laminar airflow). Dehydration might concentrate pro‐oxidants while simultaneously increasing viscosity, potentially slowing free radical chain reactions by hindering molecular diffusion at later stages (Rahmani‐Manglano et al. 2023). The controlled temperature (3°C) simultaneously inhibited microbial proliferation while permitting ongoing enzymatic and oxidative reactions. The stabilization of TBARS after Day 27 likely reflected depletion of oxidizable PUFA substrates (Pretto et al. 2024) or formation of secondary oxidation products less reactive in TBARS assays (Thamsborg et al. 2025).

### 3.3 Color Parameters Evaluation

Color stability in dry‐aged fish is a critical quality attribute influenced by moisture loss, protein concentration, and oxidative processes. Color parameters evolved significantly during aging (*p* < 0.05, Table 2), reflecting these biochemical transformations.

**TABLE 2:** Color values of dry‐aged dorsal epaxial muscle of mahi‐mahi (*Coryphaena hippurus*) (*n* = 6).

Aging day	*L*	a*	b*	C*	*h*
D0	50.10^b^ ± 3.68	0.72^a^ ± 2.64	10.49^a^ ± 3.33	10.76^a^ ± 3.42	−0.01^a^ ± 1.48
D15	53.95^a^ ± 1.71	−2.49^c^ ± 1.15	7.99^b^ ± 1.92	8.50^b^ ± 1.57	−1.24^a^ ± 0.19
D27	44.30^c^ ± 5.36	−1.81^c^ ± 1.23	8.38^b^ ± 2.01	8.69^b^ ± 1.77	−0.81^a^ ± 1,15
D42	39.44^d^ ± 4.26	−0.34^b^ ± 0.81	5.62^b^ ± 0.62	5.68^c^ ± 0.57	−0.46^a^ ± 1.51

*Note*: Different letters in the same column means significant differences between aging days by Tukey's HSD post‐hoc test (*p* < 0.05) followed by ±SD.

Lightness (L*) decreased markedly (*p* < 0.05) from 50.10 (Day 0) to 39.44 (Day 42), correlating with progressive moisture loss (76.9% to 61.5%), directly correlating with progressive moisture loss (76.9% to 61.5%, Section 3.1; *r* = 0.94, *p* < 0.05). This surface darkening resulted in increased light absorption by a denser protein matrix as water was removed (Adeyeye 2019). A similar phenomenon is also observed in dry‐aged beef due to moisture evaporation and myoglobin concentration (Bulgaru et al. 2022).

Yet, a* color parameter shifted (*p* < 0.05) from slightly positive (0.72 at Day 0) to negative values (−0.34 at Day 42), indicating a transition toward greenish tones characteristic of myoglobin oxidation to metmyoglobin. This color change linked to two concurrent processes increased protein content (from 20.9% to 35.4%), suggesting protein denaturation exposed heme groups to pro‐oxidant conditions (Ribeiro et al. 2021) and peak lipid oxidation at Day 27 (0.56 mg MDA/kg), as lipid peroxides are known to interact with and accelerate myoglobin oxidation (Zheng et al. 2016).

The b* color parameter followed a non‐linear trend, peaking at Day 27 (8.38) before declining to 5.62 at Day 42 (*p* > 0.05). The initial increase coincided with peak TBARS values and maximum oleic acid release (Section 3.5), suggesting Maillard reaction products and secondary lipid oxidation compounds (e.g., MDA) contributed to yellow‐brown hue development (Steppeler et al. 2016). The subsequent decline suggested breakdown of these pigments into colorless or polymeric compounds under prolonged oxidative stress (Bulgaru et al. 2022), consistent with sustained high TBARS levels beyond Day 27 (0.53–0.56 mg MDA/kg), (*p* > 0.05).

As well, chroma (C*) fluctuated throughout aging (*p* < 0.05), indicating preserved overall color intensity despite individual parameter shifts. However, the near‐zero hue angle (*h°*), masked by redness dominance in calculations, belied the subtle shift toward green tones due to negative a*. This discrepancy underscored the importance of evaluating all CIELab parameters collectively, as relying solely on hue angle might obscure important oxidative changes linked to myoglobin degradation (Hernández et al. 2016).

In contrast, Takahashi et al. (2023) reported that low‐temperature heating (30°C) effectively promoted fish aging without significant discoloration (*p* < 0.05), maintaining inosine‐5′‐monophosphate content and preventing protein denaturation typically responsible for color changes. Their optimal heating time (120 minutes) improved taste and texture without adversely affecting color metrics.

### 3.4 Textural Evaluation

TPA revealed significant (*p* < 0.05) structural modifications in mahi‐mahi dorsal epaxial muscle during dry‐aging, reflecting the interplay between proteolytic softening and dehydration‐induced firming (Table 3).

**TABLE 3:** Texture profile values of dy‐aged dorsal epaxial muscle of mahi‐mahi (*Coryphaena hippurus*) (*n* = 6).

Aging day	Hardness (N)	Adhesiveness	Gumminess	Chewiness (N)
D0	4179.84^a^ ± 1141.18	−31.00^a^± 12.58	1351,12^a^ ± 31.02	577.85^a^ ± 141.53
D15	997.18^c^± 249.12	−63.87^b^ ± 7.17	554.68^c^± 123.43	393.92^b^ ± 78.63
D27	2250.50^b^ ± 1317.60	−159.77^d^ ± 31.37	1067.50^b^ ± 362.50	433.33^b^ ± 144.60
D42	1921.40^b^ ± 300.93	−97.39^c^ ± 16.19	1020.82^b^ ± 153.31	575.68^a^ ± 100.18

*Note*: Different letters in the same column means significant differences between aging days by Tukey's HSD post‐hoc test (*p* < 0.05) followed by ±SD.

Hardness decreased sharply (*p* < 0.05) from 4179.84 N at Day 0 to 997.18 N at Day 15, representing a 76% reduction, which indicated rapid early‐stage softening due to proteolytic breakdown of myofibrillar proteins (Teixeira et al. 2022). This softening directly correlated with the period of minimal lipid oxidation (0.21 mg MDA/kg, Day 15) and preceded significant fatty acid changes (Section 3.5), confirming that proteolysis, primarily via calpains and cathepsins, dominated the initial aging phase independently of oxidative processes (Zhong et al. 2025). Thereafter, hardness partially recovered, increasing to 2250.50 N at Day 27 and 1921.40 N at Day 42 (*p* < 0.05 vs. Day 15). This increase likely reflected moisture reduction (Table 1) increasing protein density, partially offsetting enzymatic tenderization (Permatilleke et al. 2022). Similar dehydration‐induced firming counteracted proteolytic softening in dry‐aged beef (Bulgaru and Popescu 2023).

Adhesiveness (stickiness) decreased over time, shifting from −31.00 N·s (Day 0) to −159.77 N·s at Day 27 (*p* < 0.05), indicating increased surface dehydration and reduced water‐binding capacity. This trend temporally aligned with both moisture loss and the onset of significant lipid oxidation at Day 27, suggesting that protein denaturation was potentially exacerbated by oxidative cross‐linking and exposed hydrophobic residues while reducing surface moisture (Ribeiro et al. 2021; Stankevich et al. 2024). The slight rise at Day 42 (−97.39 N·s, *p* < 0.05) might reflect structural reorganization of muscle fibers as dehydration progressed beyond 35% moisture loss, potentially forming new inter‐protein interactions that modified surface properties, which is explained by progressive dehydration reducing surface moisture and minimizing sticky protein interactions (Permatilleke et al. 2022).

Gumminess, which means energy required to disintegrate the sample, followed a similar trend to hardness, declining from 1351.12 N (Day 0) to 554.68 N at Day 15 (*p* < 0.05), then partially recovering to 1067.50 N at Day 27. Also, chewiness, the work needed for mastication, decreased significantly from 577.85 N·(Day 0) to 393.92 N·at Day 15 (*p* < 0.05), correlating with early proteolytic softening, then increased to 575.68 N·mm by Day 42 (*p* < 0.05). However, a significant increase to 575.68 N·occurred by Day 42 (*p* < 0.05), suggesting that dryness‐induced firming counteracted proteolytic softening in later stages, consistent with moisture loss and protein aggregation (Permatilleke et al. 2022).

This texture behavior, as rapid softening followed by partial firming, reflected a kinetic competition between enzymatic tenderization and dehydration‐induced protein aggregation (Barajas Gamboa et al. 2025). Fish muscle undergoes faster proteolysis than mammalian meat due to higher cathepsin activity, explaining the pronounced early softening (Stankevich et al. 2024). The subsequent firming coincided with peak oxidation and may involve oxidative cross‐linking of proteins, further linking texture changes to the broader oxidative cascade documented throughout this study.

### 3.5 Evaluation of Fatty Acid Profile During Dry‐Aging

The fatty acid profile of mahi‐mahi dorsal epaxial muscle underwent significant modifications during dry‐aging (*p* < 0.05, Table 4), reflecting the combined effects of enzymatic lipolysis and oxidative reactions, which are key processes influencing nutritional quality.

**TABLE 4:** Fatty acid profile and nutrition quality index values of dry‐aged dorsal epaxial muscle of mahi‐mahi (*Coryphaena hippurus*) (*n* = 6).

Fatty acid/aging day	0	15	27	42
C4	5.52^a^±0.58	5.12^a^±0.04	4.64^a^±0.67	4.77^a^±0.04
C6	4.31^a^±0.47	3.65^a^±0.12	3.30^a^±0.06	3.76^a^±0.20
C8	2.47^a^±0.37	1.98^a^±0.06	1.85^a^±0.02	2.14^a^±0.10
C10	4.75^a^±0.39	4.20^a^±0.01	4.05^a^±0.16	4.43^a^±0.13
C12	2.92^a^±0.98	3.03^a^±0.06	2.87^a^±0.27	3.23^a^±0.31
C14	12.46^a^±1.09	11.36^a^±0.15	11.62^a^±1.05	12.73^a^±0.28
C16	31.21^a^±2.06	27.40^a^±0.12	25.36^b^±0.29	30.22^a^±1.58
C18	17.10^b^±1.17	15.76^b^±0.42	15.62^b^±0.33	19.25^a^±1.01
*ω*6	1.27^a^±0.62	1.22^a^±0.03	1.46^a^±0.01	0.90^a^±0.31
*ω*9	17.87^b^±2.30	26.12^a^±0.05	30.00^a^±1.55	18.55^b^±0.91
AI	4.39^a^±0.87	2.77^b^±1.04	2.36^b^±0.76	4.34^a^±0.93
TI	4.56^b^±1.32	3.99^c^±1.18	3.34^c^±0.98	6.40^a^±1.17
H/H	0.44^b^±0.27	0.70^a^±0.14	0.85^a^±0.23	0.42^b^±0.08

*Note*: Different letters in the same row means significant differences between aging days by Tukey's HSD post‐hoc test (*p* < 0.05) followed by ±SD.

Abbreviations: AI, atherogenic index; TI, thrombogenic index; H/H, hypocholesterolemic/hypercholesterolemic fatty acid ratio.

Short‐ and medium‐chain fatty acids (C4:0–C14:0) remained stable throughout aging (*p* > 0.05). This stability, combined with observed decreases in PUFAs, aligned with current understanding that lipolytic enzymes in dry‐aged products exhibited selectivity based on lipid class (phospholipids vs. triacylglycerols) and degree of unsaturation rather than chain length per se (Tatiyaborworntham et al. 2022; Ye et al. 2024). Palmitic acid (C16:0) decreased significantly by Day 27 (25.36% vs. 31.21% at Day 0; *p* < 0.05) and then raised to 30.22% at Day 42 (*p* > 0.05 vs. Day 0). This nonlinear pattern likely reflected initial triglyceride hydrolysis by endogenous lipases (Khetarpal et al. 2021), followed by secondary oxidation or volatilization of unsaturated fatty acids, concentrating more stable saturated fractions (Saidaiah et al. 2024). Stearic acid (C18:0) followed a different trajectory, remaining stable until a significant increase at Day 42 (19.25% vs. 17.10% at Day 0; *p* < 0.05). This relative increase is consistent with phospholipid hydrolysis releasing fatty acids from membranes (Tatiyaborworntham et al. 2022; Chao et al. 2020), coupled with selective oxidative loss of PUFAs, which are more susceptible to degradation (Tatiyaborworntham et al. 2022; Yang et al. 2022), thereby enriching the proportion of saturated stearic acid in the remaining lipid fraction (Yang et al. 2022; Di Paolo et al. 2023). Similar FFA profile alterations occur in prolonged dry‐aging meats (Zuljargal et al. 2023).

Oleic acid (*ω*‐9) increased from 17.87% at Day 0 to a peak of 30.00% at Day 27 (*p* < 0.05), before declining to 18.55% by Day 42. This pattern suggested initial dominance of lipolysis, with lipases hydrolyzing triacylglycerols and phospholipids to release free fatty acids, particularly oleic acid (Tatiyaborworntham et al. 2022). The subsequent decline aligned with increased lipid oxidation (Section 3.2), as prolonged oxidative conditions degraded even less‐susceptible monounsaturated fatty acids (Zhou et al. 2022). The stability of *ω*‐6 fatty acids likely reflected their low initial concentration, limiting detectable oxidative impact.

The stability of short‐chain fatty acids throughout aging suggested either preferential hydrolysis of longer chain triglycerides or rapid metabolism of short‐chain species, as lipid degradation in fish is influenced by fatty acid structure and susceptibility to enzymatic cleavage (Ying et al. 2025). The dynamic changes in long‐chain saturated fatty acids, particularly the mid‐aging decline in palmitic and stearic acids, reflected progressive lipolysis, as FFAs were released during storage via endogenous lipase action (Nie et al. 2022). The subsequent increase by Day 42 may indicate selective preservation of saturated fatty acids due to their lower oxidative susceptibility compared to unsaturated counterparts.

The *ω*‐9 behavior suggested selective hydrolysis of fatty acids esterified to glycerol early in aging, followed by oxidative breakdown. That process is supported by Zhou et al. (2022), who demonstrated that marine lipids, particularly those high in PUFA, undergo rapid oxidation. The relative stability of ω‐6 fatty acids compared to ω‐9 may reflect differences in oxidative susceptibility, as omega‐6 fatty acids are generally less prone to oxidation than omega‐3 or omega‐9 counterparts (Alagawany et al. 2022). These findings aligned with observations in dry‐aged beef (Zuljargal et al. 2023), confirming that dry‐aging induces characteristic lipolytic and oxidative modifications across meat species.

### 3.6 Nutrition Quality Index Evaluation

The evaluated nutritional quality indices, namely AI, TI, and fatty acid ratio H/H, revealed significant changes in lipid health quality during dry‐aging (*p* < 0.05, Table 4). These indices reflect the balance between pro‐atherogenic saturated fatty acids and beneficial unsaturated fatty acids.

AI decreased significantly from 4.39 at Day 0 to 2.36 by Day 27 (*p* < 0.05), indicating reduced cardiovascular risk potential during early to mid‐aging (Li et al. 2024; Nava et al. 2023). However, this trend reversed sharply by Day 42, with AI rising to 4.34 (*p* < 0.05 vs. Day 27), suggesting a resurgence of pro‐atherogenic saturated fatty acids (Ariunbold et al. 2023). Similarly, TI followed this pattern, decreasing progressively from 4.56 at Day 0 to 3.34 at Day 27 (*p* < 0.05), then increasing markedly to 6.40 by Day 42 (*p* < 0.05), reflecting deterioration in thrombogenic potential during extended aging (Ulbricht and Southgate 1991; Santos‐Silva et al. 2002). The H/H ratio, measuring balance between cholesterol‐modulating fatty acids, displayed an inverse relationship. It increased from 0.44 at Day 0 to 0.85 by Day 27 (*p* < 0.05), indicating improved lipid quality, and then declined to 0.42 at Day 42 (*p* < 0.05 vs. Day 27), signaling nutritional deterioration as oxidative processes depleted the unsaturated fraction (Nava et al. 2023).

These trends could be explained through underlying biochemical mechanisms. During early aging (through Day 27), lipase‐mediated hydrolysis of triglycerides likely released saturated fatty acids while relatively preserving unsaturated fractions, temporarily improving lipid quality indices. This aligned with established research on enzymatic lipid modification in post‐mortem fish muscle (Li et al. 2024). The improved H/H ratio and declining AI and TI during early aging parallel observations in fresh seafood, where high PUFA and MUFA content confer favorable indices (Nava et al. 2023). Conversely, the increase in AI and TI at Day 42 (*p* < 0.05) mirrored trends in aged animal products where saturated fatty acids dominate lipid profiles. This resurgence might reflect selective degradation of unsaturated fatty acids via oxidative rancidity (Section 3.2), concentrating more stable saturated fractions (Ariunbold et al. 2023). Additionally, palmitic acid (C16:0) is consistently the dominant SFA in fish lipids and is one of the SFAs that most strongly raises LDL‐cholesterol, AI, and TI, nutritional indices (Nava et al. 2023; Shantosh et al. 2023).

The evolution of nutritional quality indices directly reflected fatty acid profile dynamics driven by lipid oxidation. Early lipolysis improved indices by releasing health‐beneficial MUFAs and PUFAs; however, as aging progressed, oxidative processes preferentially degraded these unsaturated fatty acids, particularly PUFAs, generating lipid oxidation products while depleting essential fatty acids (Aduol et al. 2025; Ampem et al. 2022; Belhoussaıne et al. 2024). This selective oxidative loss, amplified by moisture loss concentrating all lipid fractions (Wealleans et al. 2021), caused a relative enrichment of saturated fatty acids in the remaining lipid profile. Consequently, AI and TI increased while the H/H ratio declined, reflecting the progressive deterioration of nutritional quality as a direct outcome of oxidative destruction of health‐beneficial unsaturated fatty acids (Aduol et al. 2025; Belhoussaıne et al. 2024; Bosco et al. 2022).

## 4 Conclusion

Dry‐aging mahi‐mahi (*C. hippurus*) dorsal epaxial muscle for 42 days induced significant biochemical transformations, including progressive moisture loss (to 61.5%), protein concentration (to 35.4%), and lipid oxidation peaking at 0.56 mg MDA/kg by Day 27. These changes directly influenced texture (initial softening followed by partial recovery), color (darkening and greenish tones from myoglobin oxidation), and fatty acid profiles (early lipolysis‐driven oleic acid release followed by oxidative degradation).

While extended aging beyond 30 days leads to irreversible nutritional deterioration, an optimal window of approximately 15–27 days was shown for mahi‐mahi. During this period, controlled proteolysis and lipolysis enhance texture modification, while the lipid profile remained nutritionally favorable. Day 27 represented the optimal compromise, where benefits of texture development outweigh initial oxidative damage.

These findings might have practical implications for the seafood industry. Controlled aging durations, approximately 27 days, optimized lipid profiles, balancing nutritional benefits (lower AI/TI, higher H/H) with texture enhancement. Beyond this window, oxidation compromises nutritional quality, emphasizing the need for optimized protocols or protective interventions (e.g., antioxidant treatments) to preserve health‐beneficial lipid components during extended aging.

Therefore, the study limitations included focus on a single species under fixed conditions. Further research should explore species‐specific proteolytic and lipolytic kinetics; antioxidant interventions (natural extracts and vacuum aging) to delay oxidation; and sensory evaluations correlating physicochemical changes with consumer acceptance. Such refinements would enable optimization of dry‐aging protocols for premium seafood products while preserving nutritional quality.

## Author Contributions

**Rômulo D. Lopes**: conceptualization, investigation, writing – original draft, formal analysis. **Sérgio B. Mano**: visualization, project administration, supervision. **André L. M. Souza**: methodology, visualization, supervision. **Jane S. M. Castro**: supervision, software, visualization. **Lévison C. Cipriano**: methodology, validation, formal analysis. **João P. A. D. Ultra**: methodology, validation, formal analysis. **Adriano G. Cruz**: writing – review and editing, supervision. **Renata S. L. R**.: supervision, formal analysis, methodology, visualization, validation. **Celso F. Balthazar**: writing – review and editing, visualization, data curation, software. **Eliane T. Mársico**: resources, project administration, visualization, supervision, data curation.

## Conflicts of Interest

The authors declare no conflicts of interest.
